# The Effectiveness of a Web-Based Self-Help Program to Reduce Alcohol Use Among Adults With Drinking Patterns Considered Harmful, Hazardous, or Suggestive of Dependence in Four Low- and Middle-Income Countries: Randomized Controlled Trial

**DOI:** 10.2196/21686

**Published:** 2021-08-27

**Authors:** Michael P Schaub, Marcela Tiburcio, Nora Martínez-Vélez, Atul Ambekar, Roshan Bhad, Andreas Wenger, Christian Baumgartner, Dzianis Padruchny, Sergey Osipchik, Vladimir Poznyak, Dag Rekve, Fabricio Landi Moraes, André Luiz Monezi Andrade, Maria Lucia Oliveira Souza-Formigoni, WHO E-Health Project On Alcohol And Health Investigators Group

**Affiliations:** 1 Swiss Research Institute for Public Health and Addiction University of Zurich Zurich Switzerland; 2 Department of Social Sciences in Health Ramón de la Fuente Muñiz National Institute of Psychiatry Mexico City Mexico; 3 National Drug Dependence Treatment Centre All India Institute of Medical Sciences New Delhi India; 4 Republican Research and Practice Center for Mental Health Minsk Belarus; 5 Department of Mental Health and Substance Abuse World Health Organization Geneva Switzerland; 6 Departamento de Psicobiologia Escola Paulista de Medicina Universidade Federal de São Paulo São Paulo Brazil; 7 Centre for Life Sciences Department of Psychology Pontifica Universidade Católica de Campinas Campinas Brazil

**Keywords:** alcohol, internet, public health, self-help, World Health Organization

## Abstract

**Background:**

Given the scarcity of alcohol prevention and use disorder treatments in many low- and middle-income countries (LMICs), the World Health Organization has launched an eHealth portal that includes the web-based self-help program “Alcohol e-Health.”

**Objective:**

We aimed to test the effectiveness of the Alcohol e-Health program in a randomized controlled trial.

**Methods:**

This was a two-arm, individually randomized, and controlled trial across four LMICs comparing the self-help program and a psychoeducation and internet access as usual waiting list. Participants were broadly recruited from community samples in Belarus, Brazil, India, and Mexico from January 2016 through January 2019. The primary outcome measure was change in the Alcohol Use Disorders Identification Test (AUDIT) score with a time frame of 6 months between baseline and follow-up. Secondary outcomes included self-reported numbers of standard drinks over the previous week and cessation of harmful or hazardous drinking (AUDIT score <8).

**Results:**

For this study, we recruited 1400 predominantly male (n=982, 70.1%) at least harmful or hazardous alcohol drinkers. The mean age was 37.6 years (SD 10.5). The participants were recruited from Brazil (n=587), Mexico (n=509), India (n=212), and Belarus (n=92). Overall, complete case analysis identified higher AUDIT changes in the intervention group (*B*=−4.18, 95% CI −5.42 to −2.93, *P*<.001, *d*=0.56) that were mirrored by changes in weekly standard drinks (*B*=−9.34, 95% CI −15.90 to −2.77, *P*=.005, *d*=0.28) and cessation rates for harmful or hazardous drinking (χ^2^_1_=14.56, N=561, *P*<.001). The supplementary intention-to-treat analyses largely confirmed these initial results.

**Conclusions:**

The expansion of the Alcohol e-Health program to other LMICs with underdeveloped alcohol prevention and treatment systems for alcohol use disorders should be considered after successful replication of the present results.

**Trial Registration:**

ISRCTN ISRCTN14037475; https://www.isrctn.com/ISRCTN14037475

**International Registered Report Identifier (IRRID):**

RR2-10.1111/add.14034

## Introduction

There is increasing interest in differences in patterns of alcohol use between low- and middle-income countries (LMICs) and high-income countries (HICs). Although alcohol use might be less common in LMICs, its risks are often more pronounced as mainly adults of low socioeconomic status [1] in these countries engage more in drinking of spirits to intoxication, a pattern very harmful to health [2]. Moreover, while alcohol is consumed by most middle-aged men and women in HICs, most users (83%) in LMICs are younger men [2]. Typical countries that follow these patterns, according to the World Health Organization (WHO) global status report on alcohol and health 2018 [3], are Belarus, Brazil, Mexico, and India (Table 1).

**Table 1:** Alcohol use and use disorder indicators for the four low- and middle-income countries of interest in 2016, according to the World Health Organization global status report on alcohol and health 2018 [3].

Country	Consumption of spirits as a proportion (%) of total alcoholic beverages recorded per capita in the last 12 months^a^	Heavy episodic drinking in the last 30 days (%)^a^		Alcohol use disorder prevalence (%)^b^	
		Male	Female	Male	Female
Belarus	49.0	40.5	12.2	33.9	6.2
Brazil	34.0	32.6	6.9	6.9	1.6
Mexico	20.0	30.6	6.1	4.3	0.4
India	92.0	28.4	5.4	9.1	0.5

^a^Age 15+ years.

^b^12-month prevalence estimates including alcohol dependence and harmful use of alcohol (age 15+ years).

There are insufficient studies summarizing treatment coverage and treatment demand for alcohol use disorders in LMICs. In a systematic review, only five of the 84 low-income countries or LMICs (applying the World Bank classification) provided sufficient epidemiological data regarding harmful alcohol use [1]. According to the World Mental Health Survey, failure and delays in treatment for substance use disorders were significantly greater in LMICs compared to HICs [4]. Treatment in LMICs mainly focuses on tertiary care services for alcohol dependence with often poor outcomes [5]. Research among brief alcohol interventions in LMICs suggests that brief interventions, mainly motivational interviewing (MI) following positive screening with a standardized instrument, can help reduce self-reported hazardous or harmful alcohol use in primary care populations [6]. However, implementing brief alcohol interventions in practice and scaling them up is challenging, given a number of factors like fear of stigmatization for the patient, time pressure on the practitioner, and lack of funding for adequate training at the system level [7], which makes internet-based interventions so attractive [8].

Internet interventions that target harmful or hazardous alcohol use have been developed for HICs. In meta-analyses, the largest effect sizes were reported for studies integrating different treatment principles such as cognitive behavioral therapy (CBT), principles of self-control (PSC), and personalized normative feedback (PNF) [9,10]. These studies were superior to interventions employing elements of PNF as stand-alone interventions [9]. As expected, the first cost-effectiveness studies on internet programs for alcohol abuse were very positive. A study in a treatment center demonstrated the superiority of internet-based therapy over internet-based self-help regarding value for money when considering quality-adjusted life years gained [11]. Access to an alcohol reduction website via a brochure resulted in less costs with no worse outcome when compared to a standard face-to-face brief intervention in primary care settings [12]. However, there is a paucity of research among internet-based preventative and treatment self-help programs targeting hazardous or harmful alcohol use and alcohol use disorders [9] and mental health in general [13] in LMICs.

On December 6, 2012, the WHO launched the WHO eHealth portal for alcohol and alcohol-related consequences on health, as part of activities to reduce hazardous and harmful drinking in populations, following the objectives of the WHO’s global strategy to reduce the harmful use of alcohol [14]. The portal includes the web-based self-help program called “Alcohol e-Health,” an evidence-based intervention initially developed in the Netherlands [15] as a means to reduce harmful or hazardous alcohol use and use suggestive of dependence. This program has been completely revised and implemented by the WHO Department of Mental Health and Substance Use, with institutes and organizations in Belarus, Brazil, India, and Mexico. The revised intervention’s effectiveness was tested in a randomized controlled trial (RCT) across the four involved countries [16]. The study’s primary hypothesis was that Alcohol e-Health program participants would exhibit greater reductions in their Alcohol Use Disorders Identification Test (AUDIT) score (primary outcome [17]) between baseline and a 6-month follow-up than control subjects allocated to psychoeducation and access to the internet as usual.

## Methods

### Design

This study compared the Alcohol e-Health program, based on CBT, MI, and PSC, with an assessment, psychoeducation, and access to the internet as usual control group for reducing alcohol use disorder (AUDIT score) in an individually randomized controlled four LMIC trial.

The trial was executed in compliance with the Helsinki Declaration, and approved by the WHO Ethics Review Committee in October 2015 (RPC756) and four country-specific ethics committees. The study has been registered at Current Controlled Trials (registration number: ISRCTN14037475), and the detailed study protocol was published on October 26, 2017 [16].

### Participants and the Inclusion and Exclusion Criteria

Participants were broadly recruited in community samples from Belarus, Brazil, India, and Mexico via information flyers and newspapers, magazines, radio, social media, websites, and informational events related to alcohol and health from January 2016 through January 2019. This broad recruitment strategy allowed for different recruitment conditions in the participating countries. However, there were considerable delays initiating recruitment in India due to official study approval procedures and changes in the study team. Moreover, continuous recruitment in Belarus proceeded very slowly. The study inclusion and exclusion criteria, and the rationale behind them are summarized in Table 2.

**Table 2:** Overview of the study inclusion and exclusion criteria, and the rationale behind them.

Criteria		Rationale
**Inclusion criteria**		
	Age between 18 and 75 years	To ensure a minimal age of participation
	A resident of one of the participating pilot countries	To be covered by local ethics board approval
	At least weekly internet access	To ensure at least minimal program access
	A screening AUDIT^a^ score ≥8	To include adults with potentially hazardous or harmful alcohol consumption, and those whose drinking habits are suggestive of dependence
**Exclusion criteria**		
	Current substance abuse treatment	To avoid confounding treatment effects
	Use of opioids, inhalants, cocaine/crack or amphetamine/amphetamine-like stimulants, sedatives over the past month, or cannabis/synthetic cannabinoids for more than 4 days over the past month	To prevent confounding effects with other frequently used mind-altering drugs

^a^AUDIT: Alcohol Use Disorders Identification Test.

### Intervention and Comparator

Subjects in the active study arm participated in the Alcohol e-Health program, while controls were assigned to a “waiting list,” where they were offered general information on alcohol and its effects on health, and access to the internet as usual. Program access was granted 6 months later.

The Alcohol e-Health program is an accessible interactive self-help tool for people seeking to reduce or discontinue their use of alcohol (Multimedia Appendix 1). Participants can register and use the program in their own time and free of charge. Alcohol e-Health provides support to encourage individuals to think about their drinking habits, decide whether to change their drinking behaviors, set goals regarding their drinking, take action toward reducing or stopping their drinking, measure their progress, and deal with relapses.

The core element of the program was a comprehensive diary, where participants could record every consumption occasion in terms of when, where, what, and how much they drank; with whom and how they feel about it; and other comments. Consumption of alcoholic beverages was measured in standard drinks as per the WHO Audit definition (10 g of alcohol), and a calculator assisted participants to convert their drinks into standard drinks. The drinking diary was filled out daily by dragging and dropping icons representing country-specific common drinks. Furthermore, participants could set goals for maximum number of standard drinks each day. Diary data were used for tailored weekly feedback with respect to meeting these drinking goals. Feedback was generated automatically based on individual drinking goals and country standards for low-risk drinking.

After having set baseline benchmarks regarding drinking, participants could explore the advantages and disadvantages of drinking and subsequently analyze their motivation to change. Furthermore, they were encouraged to identify risky situations with suggestions to deal with them and motivational strategies to help them maintain higher levels of resistance under such circumstances (development of alternative action plans). A relapse module allowed participants to analyze these situations (where and when, with whom, thoughts, personal feelings and thoughts, and consequences) and to cope with potential relapse (development of alternative helpful thoughts and actions in these situations). Graphical summaries of entries supported users to quickly identify core elements of risky situations. In the persistence module, users explored how they could resist social pressures to drink excessively. To address technical problems that occurred during their participation in the program, participants were permitted to contact, by email in their native language, a technician qualified to deliver technical assistance. A detailed program description is provided in the study protocol paper (Multimedia Appendix 2) [16].

Conversely, those within the psychoeducation and internet access as usual control group were told that they would be provided access to the program in 6 months, and referred to a web page containing information about the various types of alcoholic beverages, standard drink definitions, effects of alcohol on the mind and body, social effects of drinking alcohol, risk factors for alcohol dependence, women and alcohol, and adolescent alcohol use.

Throughout the 6-week program, all participants were encouraged to see a health professional if they experienced acute alcohol withdrawal or other severe physical or mental symptoms, and were afforded access to a country-specific medical advisory and emergency list.

### Measurement Instruments

The main outcome was change in the adjusted AUDIT [17] score between baseline and a 6-month follow-up. Corresponding AUDIT versions were provided in English, Portuguese, Russian, and Spanish. Since the follow-up period was limited to 6 months, the AUDIT was assessed for the last 6 months instead of the last 12 for items 9 and 10, both at baseline and follow-up [17].

Secondary outcomes were as follows (Table 3): (1) falling below the cutoff of hazardous or harmful alcohol use (AUDIT score <8); (2) weekly number of standard drinks (based on a single question with seven answering fields asking about alcohol use, in standard drinks, on each day of a typical week); and (3) program satisfaction, rated using the 8-item Client Satisfaction Questionnaire (CSQ-8) [18] (assessed 6 weeks after baseline). At the 6-month follow-up, participants were asked to grade any negative effects they had experienced, as per the report by Rozental et al [19], and if they had received any external help.

**Table 3:** Overview of the study measurements.

Assessments/instruments	Baseline^a^	Week 6 follow-up	Month 6 follow-up^a^
Sociodemographics	Yes	No	No
AUDIT^b^ score	Yes	No	Yes
Weekly number of standard drinks^c^	Yes	No	Yes
8-item Client Satisfaction Questionnaire	No	Yes	No
Adverse effects	No	No	Yes

^a^Where “Yes” is indicated both at baseline and the 6-month follow-up, the outcome of interest is the change between baseline and the 6-month follow-up.

^b^AUDIT: Alcohol Use Disorders Identification Test.

^c^Based on a single question with seven answering fields asking about alcohol use, in standard drinks, on each day of a typical week.

### Sample Size

The initial sample size estimate (Cohen *d*=0.40 [14], 95% confidence [α=.05], and 95% power [1−β=0.95]) for analysis of covariance with one covariate (country) was 708 overall, when controlling for cluster effects [16]. However, as we met considerable follow-up problems in Brazil (see Multimedia Appendix 3 for country-specific trial flows) and as study implementation in India was delayed for technical reasons, we started to recruit only in Brazil and Mexico, with over-recruitment by 50% for the increased missing follow-up data (achieved N with 52% over-recruitment for Brazil and Mexico, including some data from Belarus [n=92], equaling 1188). However, recruitment started in India in January 2019 (n=212). Finally, the study team decided to include participants from India to increase validity for a LMIC. This resulted in a total N of 1400.

### Study Procedures, Screening, and Consent

Once potential participants arrived on the Alcohol e-Health program home page, they were asked to complete the AUDIT and subsequently received personalized feedback, according to their individual drinking level concerning nonrisky drinking (<8 drinks/week), potentially harmful or hazardous drinking (8-19 drinks/week), and drinking suggestive of dependence (≥20 drinks/week). Those with an AUDIT score ≥8 were given details about the study, including (1) study aims and duration; (2) inclusion and exclusion criteria (Table 2); (3) two different study conditions; (4) potential risks of participation and safety agreements; (5) information that Alcohol e-Health cannot replace face-to-face interventions; (6) information on how participation was entirely voluntary and on their right to withdraw from the study at any time without consequences; and (7) information that the study had been approved by the WHO Research Ethics Committee and the four country-specific ethics committees. Informed consent was assumed when they selected all the necessary fields on the online informed consent form and clicked the submission icon. After having consented, participants filled out baseline questionnaires and were randomized, by a computer, to either the Alcohol e-Health program or control group, in a 1:1 ratio in each country [16]. This nonblinded assignment was registered automatically in the background database.

### Follow-Up Procedure and Compensation

The 6-month follow-up assessment was performed using a step-wise procedure including electronic follow-up and reminders, as well as telephone interviews by study collaborators in their own language. Completion of all follow-up assessments qualified subjects for participation in a raffle to win a tablet or corresponding donation to a charitable organization in each country, except Brazil owing to Ethics Committee restrictions.

### Statistical Analysis

For the baseline analysis between centers, we used chi-square analysis, analysis of variance (ANOVA), or the Kruskal-Wallis test, depending on the type of variable. The main analysis was based on complete case analyses (CCAs) and supplemented with intention-to-treat (ITT) analyses. Multiple linear models using regression analysis predicting the variable change score (baseline − follow-up) were used with baseline variables and the treatment condition as predictors. The missing data for ITT analyses were supplemented with multiple imputations and the R package MICE [20]. MICE specifies a multivariate distribution for missing data and draws imputations from their conditional distributions employing Markov chain Monte Carlo techniques. The missing data were assumed to be at random, and a total of 20 data sets were imputed in the supplemental ITT analyses. The effect sizes of the program were calculated using pooled results of linear model analysis. Sociodemographic (group, sex, age, country, and treatment center), and primary (AUDIT) and secondary (standard drinks in the last 7 days) outcome variables were included in the ITT imputation model. Treatment center was not used as a covariable in the overall analysis as the explained variance was less than 4%. Secondary analysis was based on complete cases, as the imputation of satisfaction scores and adverse effects was deemed unreasonable with the exception of the analysis of the number of standard drinks consumed in the last 7 days, which was additionally performed with imputed data.

### Dealing With Invalid Data/Outlier Data

Outlier data were removed to increase data validity, based on the number of standard drinks consumed in the last 7 days, as there were no upper limits for self-reported data. Values above three standard deviations from the mean were considered unlikely and potentially wrong; 18 data points were set to null based on this criterion. Multimedia Appendix 4 shows a sensitivity analysis with setting of the 18 data points to the maximum remaining value in the data set.

## Results

### Demographics and Baseline Characteristics

**Figure 1:**
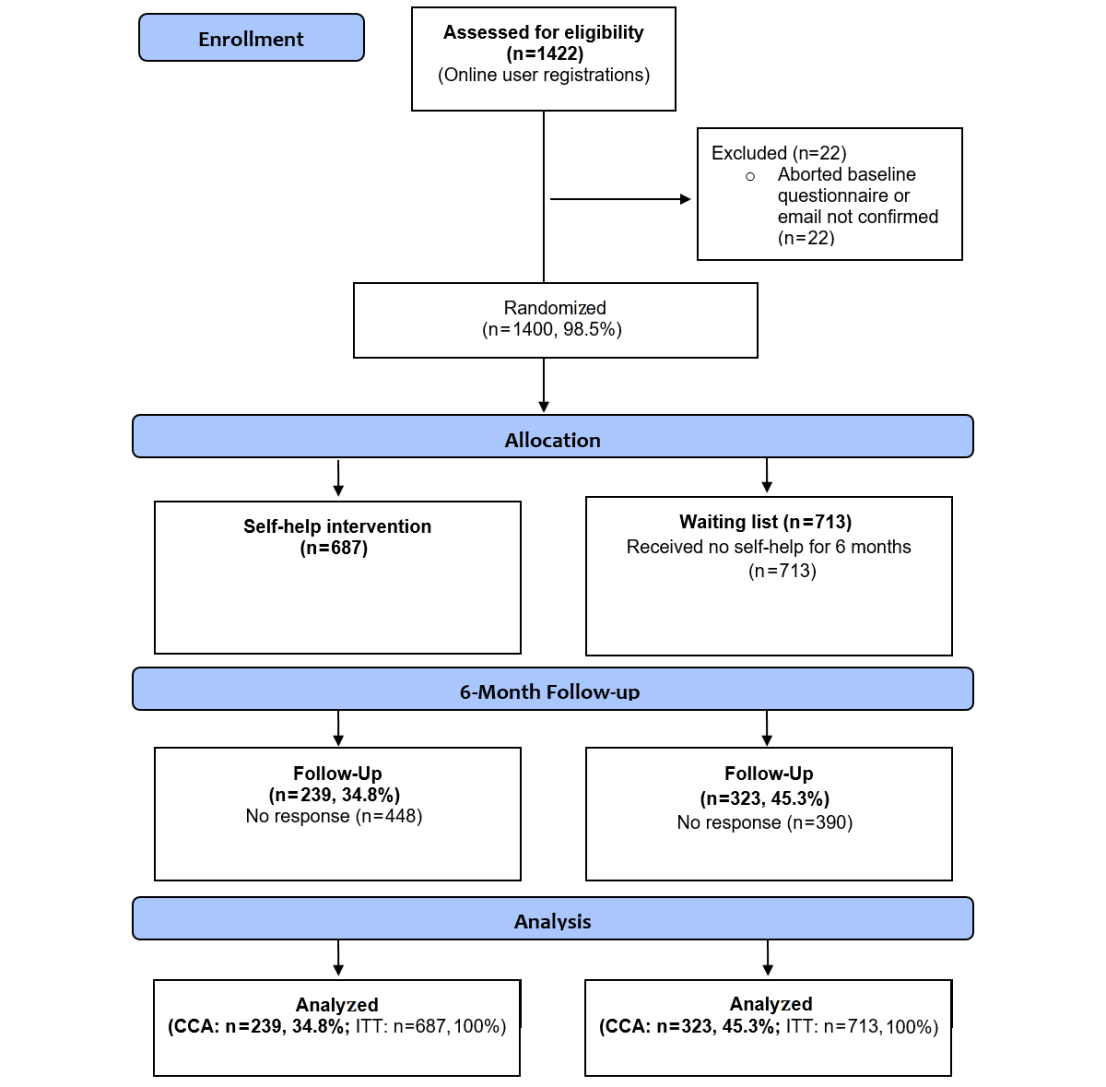
CONSORT-EHEALTH flowchart: overview of participant flow. CCA: complete case analysis; ITT: intention to treat.

Of the 1422 persons who signed up, 22 failed to complete the baseline survey. Thus, a total of 1400 participants (418 female, 29.9%) were randomly allocated to two study arms (Figure 1). Table 4 summarizes the demographic characteristics and baseline screening data of and statistical comparisons between the four study centers. The average age of participants was 37.6 years (SD 10.5). Participants consumed an average of 43.7 (SD 41.4) standard drinks per week and had an average of 2.6 (SD 2.0) alcohol-free days per week at baseline. The average AUDIT score at baseline was 23.0 (SD 7.7), with significant differences between the four centers for all variables.

**Table 4:** Demographic and baseline characteristics of all centers and study arms.

Characteristic		Brazil (N=587)		Mexico (N=509)		India (N=212)		Belarus (N=92)		Total	Statistical value^a^	*P* value
		IG^b^ (n=290)	CG^c^ (n=297)	IG (n=256)	CG (n=253)	IG (n=95)	CG (n=117)	IG (n=46)	CG (n=46)			
**Gender, n (%)**											χ^2^ (3)=68.9, N=1400	<.001
	Female	99 (34.1%)	125 (42.1%)	57 (22.3%)	82 (32.4%)	10 (10.5%)	9 (7.7%)	16 (34.8%)	20 (43.5%)	418 (29.9%)		
	Male	191 (65.9%)	172 (57.9%)	199 (77.7%)	171 (67.6%)	85 (89.5%)	108 (92.3%)	30 (65.2%)	26 (56.5%)	982 (70.1%)		
Age (years), mean (SD)		37.6 (10.6)	36.6 (10.2)	36.6 (11.0)	36.4 (10.6)	38.6 (9.5)	40.3 (8.9)	43.1 (9.4)	41.0 (10.7)	37.6 (10.5)	*F* (3)=10.4, N=1400	<.001
Alcohol Use Disorders Identification Test (AUDIT) score, mean (SD)		22.3 (6.8)	22.2 (6.5)	22.6 (6.3)	22.3 (6.8)	30.2 (7.2)	30.2 (8.6)	13.1 (4.1)	14.4 (5.7)	23.0 (7.7)	*F* (3)=142.2, N=1400	<.001
Standard drinks^d^, mean (SD)		44.6 (29.6)	42.4 (28.6)	28.3 (18.4)	30.4 (19.6)	93.0 (71.2)	90.5 (69.1)	14.5 (14.4)	13.7 (12.4)	43.7 (41.4)	*F* (3)=178.4, N=1382	<.001
Drinking-free days^d^, mean (SD)		2.8 (2.0)	2.2 (2.2)	3.2 (2.0)	2.4 (2.0)	0.4 (1.3)	0.2 (0.4)	3.0 (1.3)	2 (0.8)	2.6 (2.0)	*F* (3)=51.0, N=703	<.001

^a^Chi-square test, analysis of variance, or Kruskal-Wallis test.

^b^IG: intervention group.

^c^CG: control group.

^d^Last 7 days.

### Study Attrition and Dropout Analysis

In total, 562 (40.1%) participants completed follow-up. Dropout analysis showed a significant difference by country of enrolment (χ^2^_3_=36.88, N=1400, *P*<.001), assigned study condition (χ^2^_1_=16.11, N=1400, *P*<.001), baseline AUDIT score (*F*_1_=9.69, N=1400, *P*=.002), and baseline drink-free days over the last 7 days (*F*_1_=6.94, N=701, *P*=.009). The follow-up rate ranged from 27.6% (n=80) in the intervention group in Brazil to 56.2% (n=26) in the control group in Belarus, and in total, 34.9% (n=239) of the intervention group could be followed compared to 45.3% (n=323) of the control group. The average AUDIT score and number of drinking-free days were 22.18 (SD 7.92) and 2.35 (SD 2.07), respectively, for participants who could be followed up, and 23.48 (SD 7.49) and 2.77 (SD 2.02), respectively, for dropouts. Dropouts were not different in gender (χ^2^_1_=0.13, N=1400, *P*=.72), age (*F*_1_=2.50, N=1398, *P*=.11), baseline standard drinks over the preceding 7 days (*F*_1_=3.32, N=1380, *P*=.069), or adherence (*t*_417.5_=1.76, *P*=.08). Detailed follow-up rates are listed in Multimedia Appendix 3.

### Main Effects

Table 5 shows the detailed outcome analysis of changes in the AUDIT score (main outcome) and changes in the number of standard drinks in the last 7 days across all countries. Regression analysis based on the complete case data showed a significant decrease in the AUDIT score in the intervention group (mean 7.4, SD 7.8) compared with that in the control group (mean 3.2, SD 7.1) (*B*=−4.18, 95% CI −5.42 to −2.93, *P*<.001, *d*=0.56). Table 6 shows detailed values between baseline and follow-up from complete cases, and Multimedia Appendix 5 and Multimedia Appendix 6 show values from supplemental imputed data that confirmed the CCA results.

**Table 5:** Regression analysis results.

Variable	Intervention versus control after 6 months (complete cases) (N=562)		
	*B* ^a^	95% CI	*P* value
AUDIT^b^	−4.18	−5.42 to −2.93	<.001
Standard drinks^c^	−9.34	−15.90 to −2.77	.005

^a^Baseline data and condition as predictors for group effect.

^b^AUDIT: Alcohol Use Disorders Identification Test.

^c^Last 7 days.

**Table 6:** Values between baseline and follow-up from complete cases.

Variable	Control baseline (n=713), mean (SD)	Intervention baseline (n=687), mean (SD)	Control followed up^a^ (n=325), mean (SD)	Intervention followed up^a^ (n=239), mean (SD)	*d* ^b^	95% CI
AUDIT^c^	23.05 (7.88)	22.86 (7.50)	18.71 (9.28)	15.15 (9.06)	0.56	0.38-0.72
Standard drinks^d^	44.21 (41.70)	43.23 (41.13)	23.73 (26.32)	12.46 (16.31)	0.28	0.08-0.46
CSQ-8^e^	N/A^f^	N/A	18.92 (4.65)	21.56 (4.11)	0.60	0.40-0.79

^a^6 months after baseline (complete cases).

^b^Effect size Cohen *d* based on differences between the intervention and control groups.

^c^AUDIT: Alcohol Use Disorders Identification Test.

^d^Last 7 days.

^e^CSQ-8: 8-item Client Satisfaction Questionnaire.

^f^N/A: not applicable.

### Secondary Outcomes

#### Standard Drinks

There was a significantly higher decrease in standard drinks in the intervention group (mean 24.7, SD 39.6) compared with that in the control group (mean 15.4, SD 28.4) (*B*=−9.34, 95% CI −15.90 to −2.77, *P*=.005, *d*=0.28). Results were confirmed in the supplemental ITT analysis (see Multimedia Appendix 5 and Multimedia Appendix 6).

#### Harmful or Hazardous Alcohol Use

A total of 102 (18.2%) participants had a total AUDIT score below 8 after 6 months and could therefore be classified as no more harmful or hazardous alcohol use. There was a significant difference in the assigned group, with 57 (25.6%) in the intervention group versus 41 (12.7%) in the control group achieving a score below 8 after 6 months (χ^2^_1_=14.56, N=561, *P*<.001).

#### CSQ-8

Participants in the intervention group were significantly more satisfied with their study participation (mean CSQ-8 score 21.56, SD 4.1) compared with those allocated to the control group (mean score 18.92, SD 4.7) (*t*_415.39_=6.18, *P*<.001).

#### Adherence

Of the 687 participants in the intervention group, 258 (37.6%) had at least one diary entry, 159 (23.1%) completed the tools to maintain their targets for alcohol consumption and to resist social pressure, and 41 (6.0%) completed the relapse tool.

#### Adverse Effects

A total of 188 participants completed the Rozental adverse side effects questionnaire [17]. Of these, 136 (71.9%) answered that they had not experienced any negative effects during the study, while 33 (17.5%) claimed that an adverse effect had affected them “somewhat negatively,” 7 (3.7%) claimed “quite negatively,” and 13 (6.9%) claimed “to a great extent.” There was no significant intergroup difference (*t*_151.93_=1.35, *P*=.18).

## Discussion

### Principal Findings

To our knowledge, this is the first RCT to investigate the effectiveness of an international web-based self-help program for at least harmful or hazardous drinkers in LMICs. The observed changes in AUDIT scores (main outcome) were mirrored by all of the secondary outcomes in the CCA and mostly confirmed in the supplemental ITT analyses. Adverse effects credited to the intervention were few. Thus, expanding this program to other LMICs with underdeveloped alcohol prevention and alcohol use disorder treatment systems should be considered after successful replication of these initial findings. The achieved effect strength for ITT changes in the weekly number of standard drinks (*d*=0.27) in this study is only slightly smaller than that reported for meta-analyses from HICs (*d*=0.40) [9] and evaluation of the initial Dutch program (*d*=0.40) [15] from which development of the Alcohol e-Help program started [16]. However, effects on the main outcome, change in the AUDIT score, were even higher (CCA: *d*=0.55; ITT: *d*=0.51). Presumably, in this study, the standardized AUDIT scores [17] with validated translations were more appropriate as an outcome measure than the number of weekly standard drinks, despite country-specific adaptation of the standard drink examples.

The achieved effects were mainly grounded in the Brazilian and Mexican middle-income country data, but were also observed in data from India, the only low-income country involved. Moreover, the program attracted subjects with a high probability of alcohol dependence (mean AUDIT score above 22) in these countries. In India, the baseline AUDIT score was 30. Our Indian collaborators noted that recruitment was difficult in the general population and that some of the participants might have been reached through community clinic settings where recruitment posters were sometimes hung, which may partially explain their higher baseline scores. The only country where we failed to demonstrate program effectiveness was Belarus, the middle-income country with the highest spirit use and clearly the highest alcohol use disorder prevalence. Unfortunately, we only reached few Belarusian participants with, contrary to our expectations, a comparatively low level of alcohol use and very few with an AUDIT level suggestive of dependence. Our Belarusian collaborators have advised us that there is no sense of harmful and hazardous alcohol use in the general Belarusian population and spirit use is culturally still very accepted. Unless a medical doctor refers someone for inpatient detoxification, many Belarussians with high levels of spirit use still seem to feel they do not have a health problem warranting changes in their alcohol drinking behaviors. Thus, a comprehensive prevention campaign to make the Belarussian drinking population aware of potential health problems and the consequences of their drinking behaviors might be a required preliminary step before implementation of the Alcohol e-Help program or similar interventions. In addition, a cultural adaptation of the program to reflect these values and attitudes toward excessive alcohol consumption could also be important. This could also apply to other former Soviet Union regions with similar drinking cultures and, thus, limits the generalizability of our study results. Generalizability is further limited to individuals with sufficient reading and writing skills in the corresponding study languages, those with at least weekly internet access, and those without frequent use of illicit drugs.

Despite the typical gender gap in heavy episodic drinking and prevalence of alcohol use disorders in LMICs compared to HICs [1,2], the Alcohol e-Help program still reached a population that was almost 30.0% female, and dropout rates were similar in the two genders. It is possible that similar to HICs, relatively more women will be reached by online interventions compared to face-to-face interventions. A detailed analysis of the outcome predictors in the near future could clarify the relevance of gender and other predictors for the program’s effectiveness.

On one hand, the decision to over-recruit made it possible to include data from the delayed recruitment in India in this study. On the other hand, over-recruitment increases the chance of detecting an intervention effect. Since the effect strengths determined were analogous or, in the case of the main outcome, significantly higher than originally calculated, most of the results would probably have been significant even with the originally calculated sample size. For this reason, we have not subsequently adjusted our power calculation.

Study recruitment was set as broad as possible to encourage people from the respective general populations with at least harmful or hazardous alcohol consumption, who otherwise would not receive adequate support, to participate. In this way, we also wanted to optimize the study results’ generalizability. However, this strategy probably also led to recruitment distortions between the participating countries. In Brazil, for example, a large proportion of participants were recruited through television coverage, as the Brazilian media displayed great interest in the study. Conversely, in India, the only truly successful recruitment strategies were newspaper advertisements and hanging posters, since the Indian media would have reported on the study only if financially compensated.

One difficulty that arose due to the comparatively long study preparation and study phase was that the underlying content management system (CMS) was outdated and an update during the study phase in the four country-specific portals turned out to be tricky for reasons of methodological and data loss risks. Before the Alcohol e-Help program can be adapted for other LMICs and widely implemented, its content must be transferred to the latest CMS in a time-consuming process. However, measured against the public health impact potential and increasing spread of broadband internet access in LMICs [21], this seems to be a worthwhile investment.

A major limitation of this study is the high attrition rate of nearly 60%, which was larger than for studies in HICs [9]. In addition, selective attrition was a concern with higher dropout rates in the intervention group and more severe baseline AUDIT scores reported by noncompleters. These difficulties might compromise the stability and reliability of effects. Therefore, we based our results on the CCA and added the ITT analyses only supplementally. Up to which missing level and under which conditions data can still be imputed for ITT analysis is part of ongoing discussions [22,23]. Particularly for internet-based programs in the alcohol field, this discussion is still pending. Since follow-up surveys are obviously more difficult to conduct in low-income countries, this discussion must be held as soon as possible and a consensus should be reached in this regard. The transfer of successful programs from HICs should not be hindered merely by lack of consensus in these discussions. However, since we have carried out the first transfer of a program to reduce alcohol misuse to LMICs, there is certainly room for improvement to increase the follow-up rate. Higher financial compensation would be an obvious solution. However, we deliberately decided against this because we saw a risk of an imbalance, and thus, an uncontrollable confounder, between the different countries involved. Another possibility would be to link the follow-up survey with a content booster module, in which, for example, short personalized feedback using the AUDIT strategies for the long-term success of alcohol consumption reduction is addressed. A follow-up survey would then make more sense to some participants, and they would be more likely to participate. In addition, better imputation programs, especially for data sets with a high missing proportion, are hoped to be available soon.

Other limitations of this study are as follows: (1) self-reported use of standard drinks that might particularly be susceptible to retrospective bias, due to inclusion of individuals suggestive of dependence (AUDIT score ≥21); (2) skewed country distribution of participants with an underrepresentation of individuals from Belarus and India, which made some of the ITT analyses in these two countries impossible (Multimedia Appendix 7); and (3) incorrect programing of the originally planned adherence assessments [16]. As such, no adequate sensitivity analysis could be performed.

### Conclusions

Based on the results reported here, expansion of the program to other LMICs with underdeveloped alcohol prevention and treatment systems for alcohol use disorders should be considered after successful replication of the initial results.

## Appendix

Multimedia Appendix 1 Introduction video to the Alcohol e-Health program.

Multimedia Appendix 2 Detailed technical description of the Alcohol e-Health program.

Multimedia Appendix 3 CONSORT-EHEALTH trial flowchart: overview of participant flow split in study centers.

Multimedia Appendix 4 Sensitivity analysis replacing outliers with the highest remaining value.

Multimedia Appendix 5 Intention-to-treat regression analysis results.

Multimedia Appendix 6 Intention-to-treat means, standard deviations, and achieved effect sizes.

Multimedia Appendix 7 Detailed analysis for every study country.

Multimedia Appendix 8 CONSORT-eHEALTH checklist (V 1.6.1).

## Abbreviations

AUDIT: Alcohol Use Disorders Identification Test
CBT: cognitive behavioral therapy
CCA: complete case analysis
CMS: content management system
CSQ-8: 8-item Client Satisfaction Questionnaire
HIC: high-income country
ITT: intention to treat
LMIC: low- and middle-income country
MI: motivational interviewing
PNF: personalized normative feedback
PSC: principles of self-control
RCT: randomized controlled trial
WHO: World Health Organization
