# Supplementary material for: The Effectiveness of a Web-Based Self-Help Program to Reduce Alcohol Use Among Adults With Drinking Patterns Considered Harmful, Hazardous, or Suggestive of Dependence in Four Low- and Middle-Income Countries: Randomized Controlled Trial
Source: J Med Internet Res. 2021 Aug 27;23(8):e21686. doi: 10.2196/21686 (PMC8433861; doi:10.2196/21686)
Supplement: Multimedia Appendix 2 [file jmir_v23i8e21686_app2.pdf]

Alcohol e-Help Program

Product Description

Content

Introduction 2
Overview of the Intervention 2
Guided introduction 2
1) My baseline 2
2) My goals 3
3) Action 3
Self-paced intervention 4
Dashboard 4
Diary 5
Adding a situation 5
Overview of the day 6
Goals 6
Persist 6
Relapse 8
Progress 8
Notifications by e-mail 9

Introduction

The Alcohol e-Help program has been implemented by the World Health Organization Department of Mental Health and Substance Abuse in Geneva, in collaboration with the Netherlands' Trimbos Institute, and with several institutes and organizations in Belarus, Brazil, India and Mexico.

The development of the Alcohol e-Help program involved members of the target group in three different moments: 1) During preparation, by means of focus groups with health professionals and clients representing the target users in each participating country to investigate their opinions and attitudes towards this project, their Internet surfing behaviors, their specific needs and to evaluate the inclusion of different functionalities; 2) During programming, by including test sessions involving health professionals to evaluate the attractiveness and functionality of specific program elements; 3) In the pilot phase, by involving a group of target users to test usability and subjective surfing experience.

The purpose of the present document is to provide a concise but complete overview of the front-end functionalities of the Alcohol e-Help program. Complete information also for the back-end from the administrator perspective can be found in the more detailed Operation Manual (V. 10.0).

Overview of the Intervention

There are three main components of the intervention.

- **Guided introduction**: steps at the beginning usually done in one go.
- **Self-paced intervention:** various contents to be explored at any time during the 6 weeks (or as long as they wish)
- **E-Mail notification:** at various points in time the user receives e-mails

These three components are now described in detail.

Guided introduction

This process starts after successful registration of an account and is a **tightly guided**, meaning the user can only go forward and backward through the following webpages:

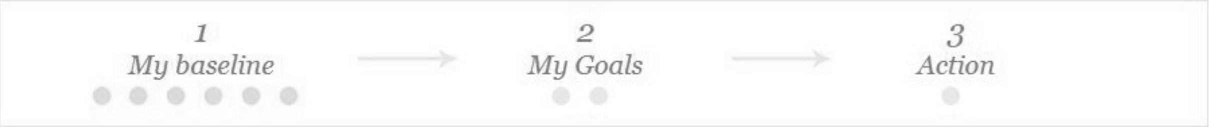

1) My baseline

a) **Alcohol Consumption of last week**
Drag & drop of various drinks into 7 bars labelled with the days of the week

b) **AUDIT**
A standardized 10 items questionnaire about alcohol use

c) **Advantages & Disadvantages of drinking**
Drag & Drop of predefined statements (with the option to add individual text) into either a box labelled "Disadvantages" or another one labelled "Advantages".

d) **Readiness to change**
A standardized 12 items questionnaire about motivation to change

e) **Summary of answers from previous 4 pages** with automatic assessment & feedback for AUDIT (Lower, moderate, high, very high risk drinking) and readiness to change.

f) **Final Self-Evaluation and decision** to go on with the program or leave.
Includes 4 yes-no questions:
- Do I drink according to the lower risk level?
- Are there reasons why I should reduce my alcohol consumption of stop drinking at all?
- What weighs heavier for me: the pros or cons from drinking?
- Am I motivated to reduce my alcohol consumption?

2) My goals

a) **Set an action plan:** Set the aimed
- number of drinks for each day of the week
- starting date
- finish date

b) **Automatic assessment and feedback** on the proposed goals according to standards of risk of drinking (lower, moderate higher or very high risk)

3) Action

a) **Introduction** of the main aspects of the following intervention.

Self-paced intervention

This process starts after the last step of the guided introduction. After this point the user is **free to navigate** through the website and explore its content at one's own pace. The following contents are structured according to the main navigation of the website:

1. Dashboard
2. Diary
3. Goals
4. Persist
5. Relapse
6. Progress
7. Evaluation

These 8 areas are now described in more detail:

Dashboard

The Dashboard is an overview area with two main components:

- Suggestions what to do next

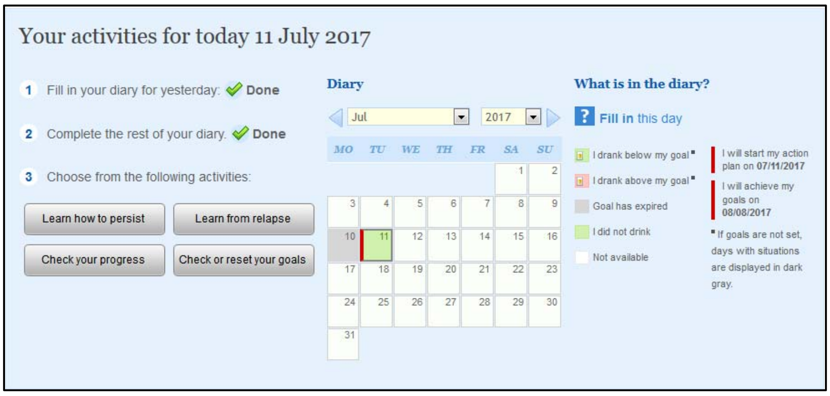

    1. Fill in the diary for yesterday
    2. Complete the rest of the diary
    3. Motivate to visit one of four pages: Goals, Persist, Relapse, Progress (see later chapters)

- Progress graph of the last seven days (see chapter Progress for further details)

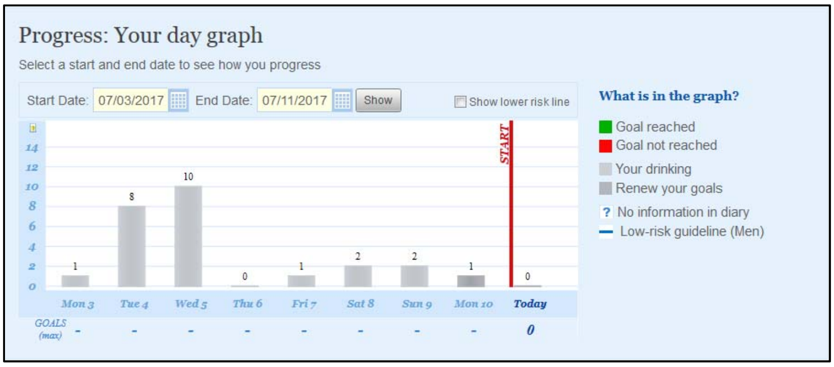

Diary

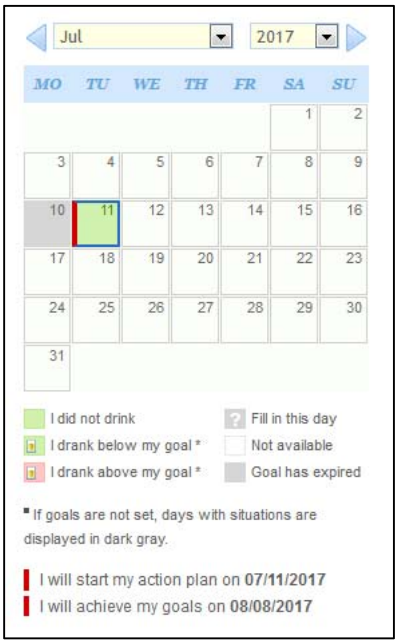

The diary offers a calendar you can browse by month and year. Every day of a month is display with a colored square indicating if the user

- did not drink (green),
- drank below the goals (green with beer icon),
- drank above the goals (red with beer icon) or
- did not yet fill out the day (grey with question mark).

Additionally a bold red stroke indicates the start and end of the action plan.

The diary is filled out by adding so called "situations". You can add multiple situations per day.

Adding a situation

When adding a situation to the diary, the user can include the following information:

- **What** drink(s) (via drag & drop of beverages)
- **When** (Morning, afternoon, evening, night, whole day)
- **How to feel about it** (Very happy, Happy, Not happy / not unhappy, Sad, Very sad)
- **Where** (home, office, pub/bar, restaurant, other)
- **With whom** (alone, spouse, family/friends, business partners, colleagues, strangers, others)
- **What feelings** (Tired, Tensed, Sad, Relaxed, Happy, Cheerful, Insecure, Pain, Other...)
- **Personal notes**

See the screenshot below to get an impression:

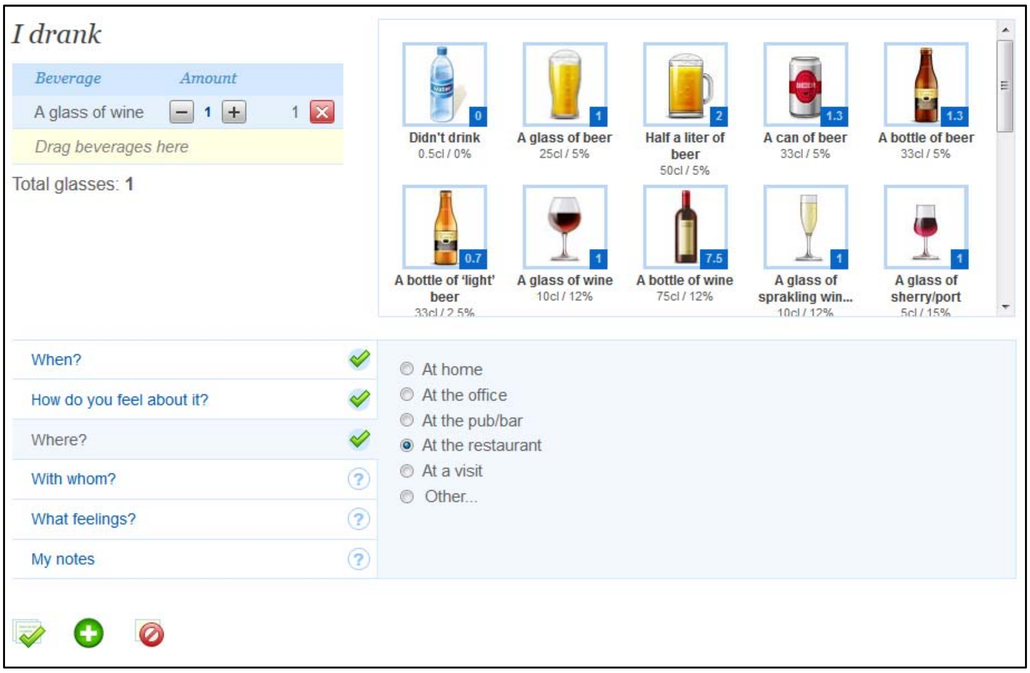

Overview of the day

The information from added situations are displayed in a user friendly way. They are ordered by the time of the day (morning, afternoon, etc). Data from ticked off boxes are combined and transformed into whole sentences. Entries can be deleted or edited. Additionally the user is always reminded on his/her goal for the current day.

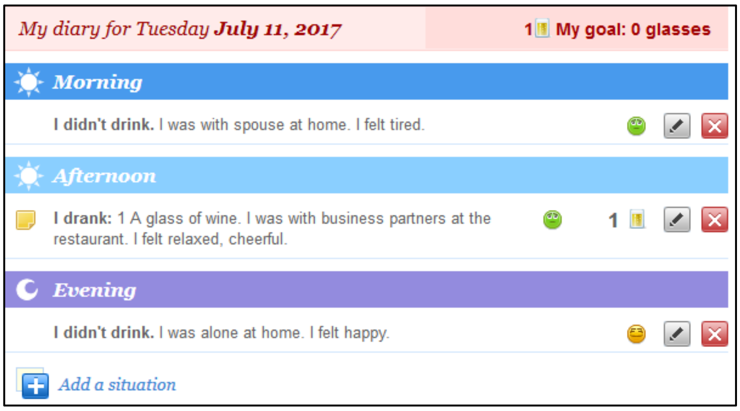

Goals

Users can revisit the goals they had set during the introduction process including the corresponding automated feedback. They can also edit those goals and set new action plans with a starting and ending date. Whenever the users reach the end of their action plan, they get notified by a pop up message asking them to set new goals.

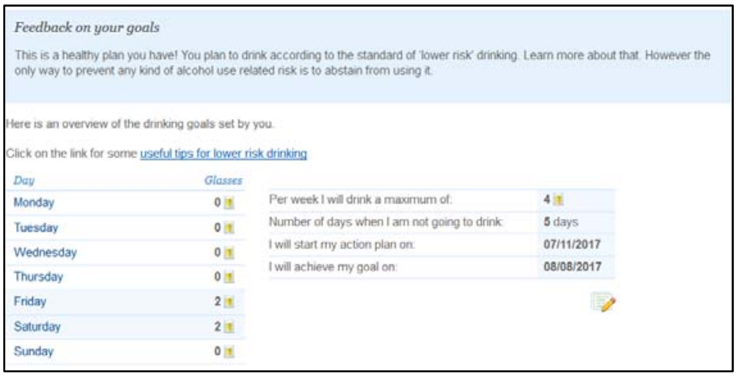

Persist

This area contains 10 topics about challenges most people face when cutting down on drinking alcohol and how to deal with them. The first half of them contain **exercises**, where the user can write down personal examples in a box.

- *Reward yourself*
- *Make a commitment with yourself*
- *How to handle risk situations*
- *Inform others*
- *Your pros and cons of not drinking or drinking less*
- *Handle social pressure*
- *Craving: Look for distraction*
- *Craving: Wait till the craving passes by*
- *Craving: Say 'stop!'*
- *Constructive thinking*

See below some example screenshots to get an impression of the exercises:

Example from “*Reward yourself*”:

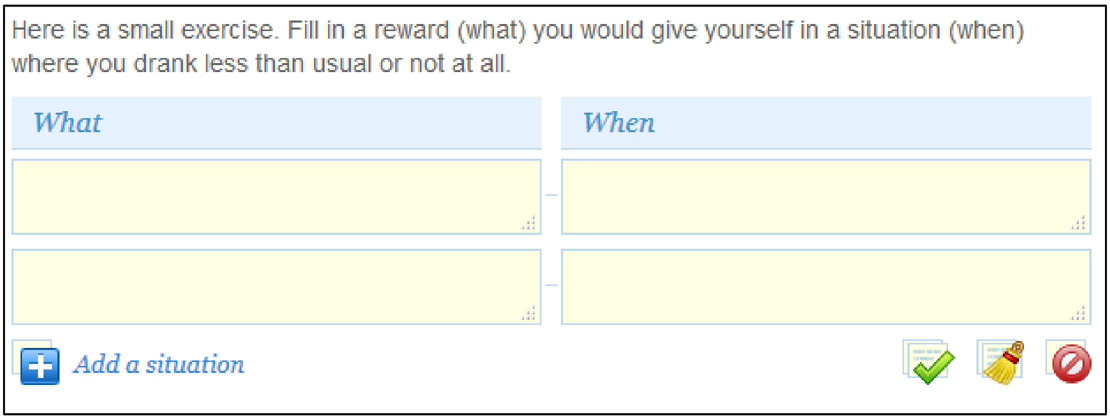

Example from “*Make a commitment with yourself*”:

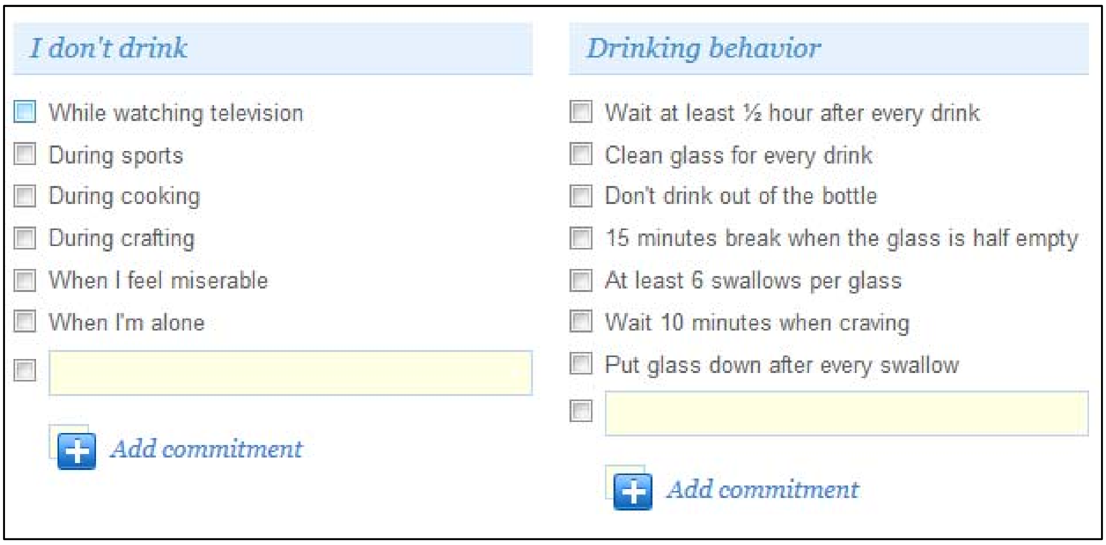

Example from “How to handle risk situations”:

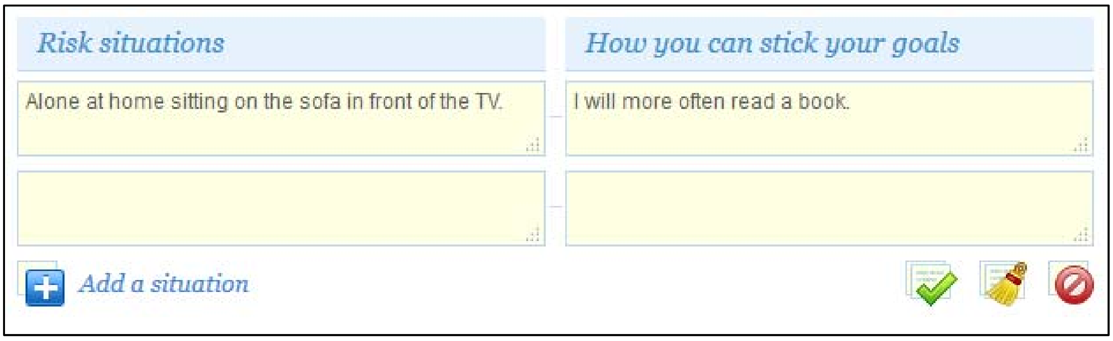

Relapse

This area contains a page on the problems of falling back into old drinking habits. It also includes an **exercise** where the user can write down

- a situation where he/she drank more than planed
- What thoughts he/she had that a) did help and b) did not
- Plan on how to deal with the situation the next time.

Progress

This area offers graphs with the number of **standard drinks per day** or **per week**. See screenshots:

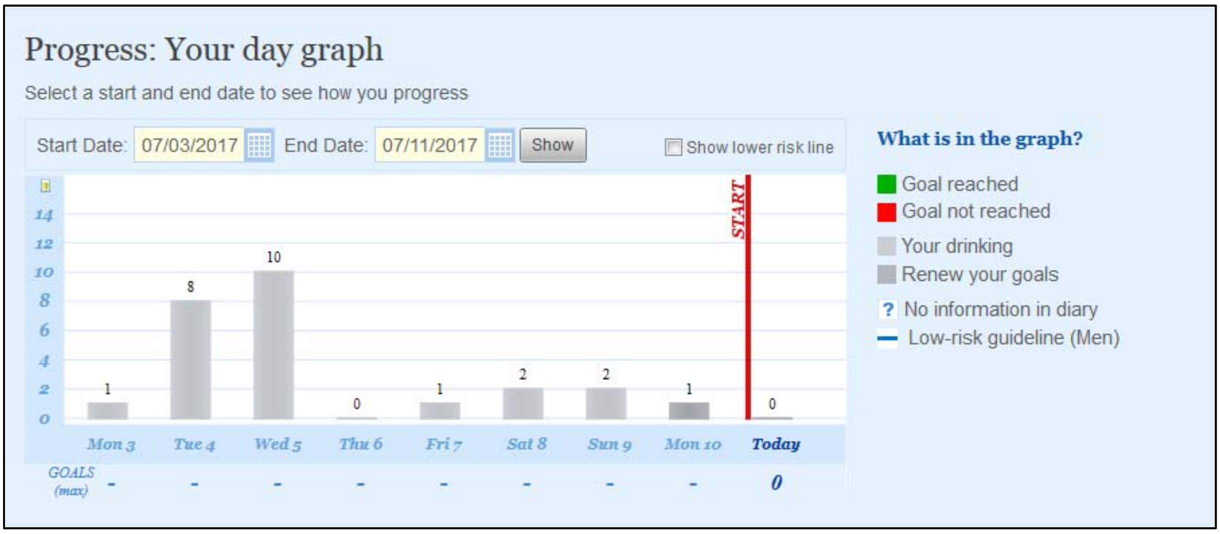

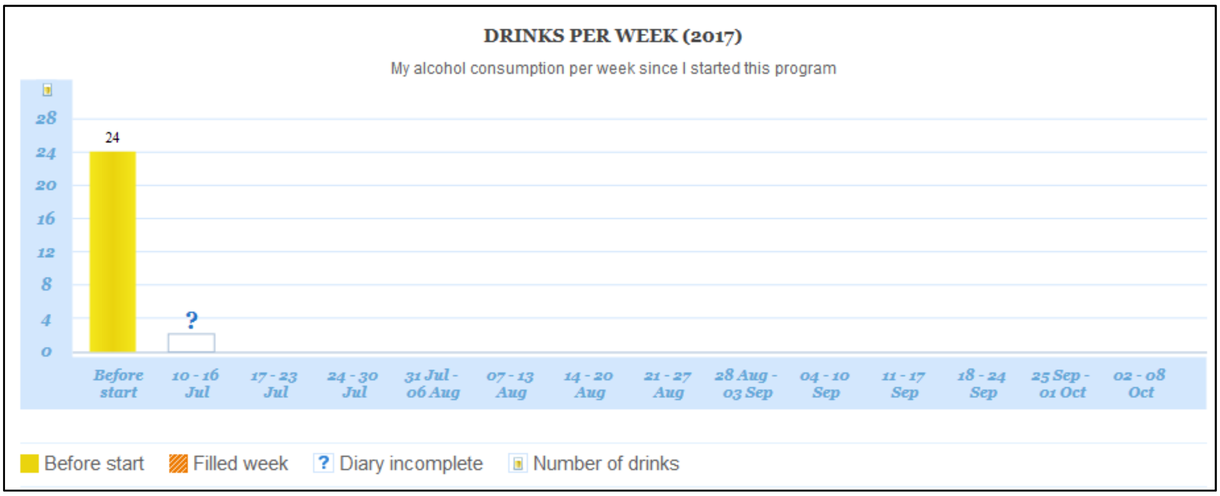

Another setting displays a graph of **days without drinking** (no screenshot).

In this area users can also revisit the feedback page from the baseline assessment during the guided introduction (1.e).

Notifications by e-mail

Users are accompanied through the intended 6 weeks by fully automated weekly emails. The main text is different for each week (addressing topics that are typical for each point in time) and also contains parts tailored to the current status of the user. Such status may be whether the user has already used the diary, the relapse prevention exercise or visited the persist area. If yes they get a praising text part, if no they get a text part encouraging them to do so.

Users also get notified if they haven't logged in for a certain amount of days.

After 6 weeks they get an email encouraging them to fill out an optional evaluation form.
