# Supplementary figures and images for: The Effectiveness of a Web-Based Self-Help Program to Reduce Alcohol Use Among Adults With Drinking Patterns Considered Harmful, Hazardous, or Suggestive of Dependence in Four Low- and Middle-Income Countries: Randomized Controlled Trial
Source: J Med Internet Res. 2021 Aug 27;23(8):e21686. doi: 10.2196/21686 (PMC8433861; doi:10.2196/21686)

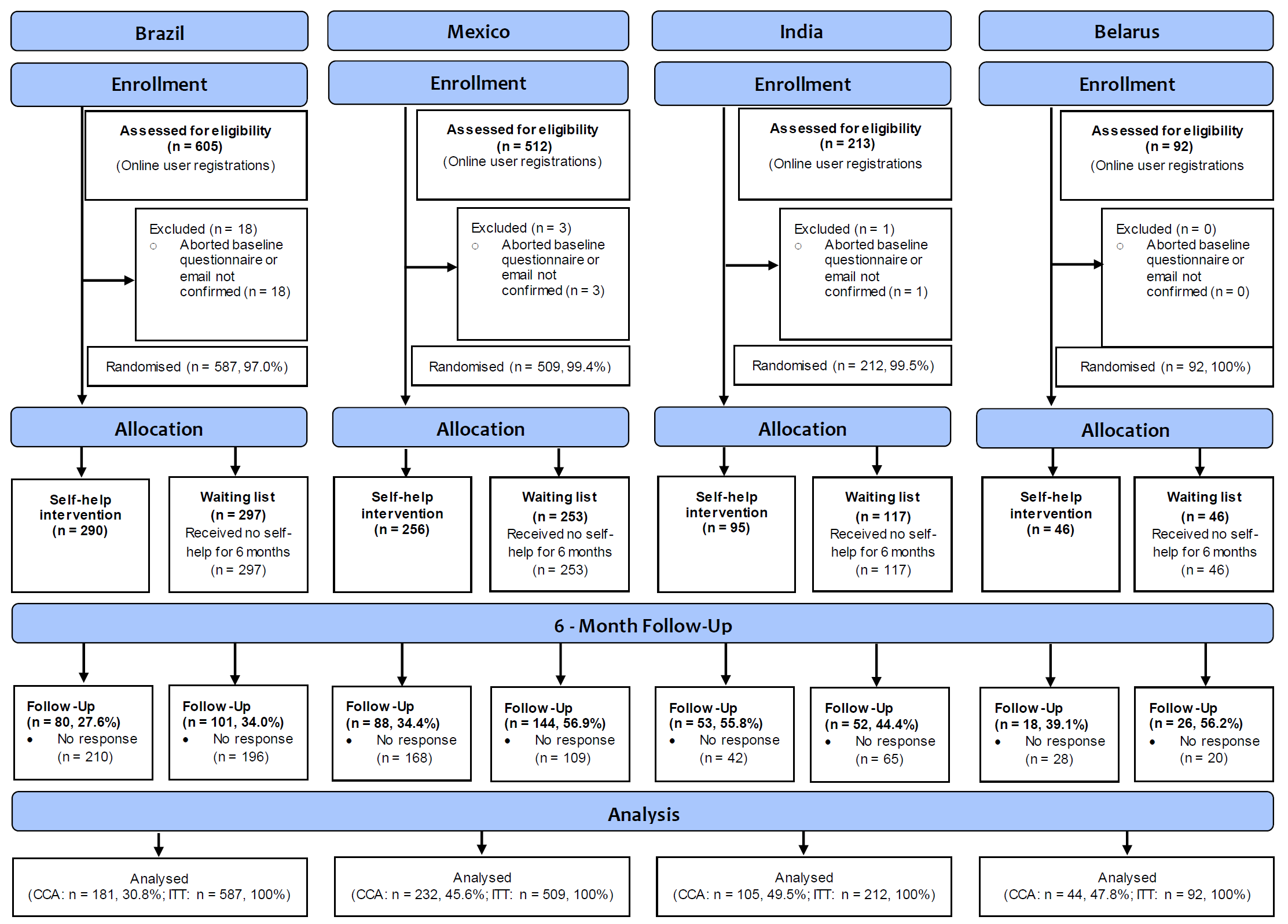

Supplement: Multimedia Appendix 3 [file jmir_v23i8e21686_app3.png]
