# Supplementary material for: The Effectiveness of a Web-Based Self-Help Program to Reduce Alcohol Use Among Adults With Drinking Patterns Considered Harmful, Hazardous, or Suggestive of Dependence in Four Low- and Middle-Income Countries: Randomized Controlled Trial
Source: J Med Internet Res. 2021 Aug 27;23(8):e21686. doi: 10.2196/21686 (PMC8433861; doi:10.2196/21686)
Supplement: Multimedia Appendix 4 [file jmir_v23i8e21686_app4.pdf]

Multimedia Appendix 4. Sensitivity analysis replacing outliers with the highest remaining value.

	Intervention versus control after 6 months (complete cases)					*Intervention versus control after 6 months (ITT analysis)*				
	B[a]	95% CI		*P*	d[c]	B[a]	95% CI		*P*	d[c]
	Followed Up (N = 562)					Imputed Data (N = 1400)				
AUDIT	-4.18	-5.42	-2.93	<.001	.56	-4.02	-5.11	-2.92	<.001	.55
Standard Drinks[c]	-7.69	-15.50	0.12	.054	.19	-8.89	-14.59	-3.19	.002	.22

ITT = Intention to Treat; AUDIT = Alcohol Use Disorders Identification Test; [a]Baseline data and condition as predictors for group effect; [b]Effect size Cohen´s d based on differences between the intervention and control group, [c]Last 7 days
