# Supplementary material for: The Effectiveness of a Web-Based Self-Help Program to Reduce Alcohol Use Among Adults With Drinking Patterns Considered Harmful, Hazardous, or Suggestive of Dependence in Four Low- and Middle-Income Countries: Randomized Controlled Trial
Source: J Med Internet Res. 2021 Aug 27;23(8):e21686. doi: 10.2196/21686 (PMC8433861; doi:10.2196/21686)
Supplement: Multimedia Appendix 5 [file jmir_v23i8e21686_app5.pdf]

**Multimedia Appendix 5.** Intention-to-treat regression analysis results.

| | *Intervention versus control after 6 months (ITT analysis)* | | | |
|---|---|---|---|---|
| | B[a] | 95% CI | | *P* |
| | Imputed Data (N = 1400) | | | |
| AUDIT | -4.08 | -5.32 | -2.85 | <.001 |
| Standard Drinks[b] | -11.61 | -17.19 | -6.03 | <.001 |

AUDIT = Alcohol Use Disorders Identification Test; [a]Baseline data and condition as predictors for group effect; [b]Last 7 days
