# Supplementary material for: The Effectiveness of a Web-Based Self-Help Program to Reduce Alcohol Use Among Adults With Drinking Patterns Considered Harmful, Hazardous, or Suggestive of Dependence in Four Low- and Middle-Income Countries: Randomized Controlled Trial
Source: J Med Internet Res. 2021 Aug 27;23(8):e21686. doi: 10.2196/21686 (PMC8433861; doi:10.2196/21686)
Supplement: Multimedia Appendix 6 [file jmir_v23i8e21686_app6.pdf]

Multimedia Appendix 6. Intention-to-treat means, standard deviations, and achieved effect sizes.

	Baseline		*6 months after baseline (ITT analysis)*				
	Mean	SD	Mean	SD	d[a]	95% CI	
Control (n = 713)			Imputed Data (n = 713)				
AUDIT	23.05	7.88	19.78	9.37			
Standard Drinks[b]	44.21	41.70	28.78	32.00			
CSQ-8							
Intervention (n = 687)			Imputed Data (n = 687)				
AUDIT	22.86	7.50	15.52	8.73	0.56	0.44	0.65
Standard Drinks[b]	43.23	41.13	15.92	20.33	0.30	0.19	0.40
CSQ-8							

ITT = Intention to Treat; AUDIT = Alcohol Use Disorders Identification Test; CSQ-8 = Client Satisfaction Questionnaire; [a]Effect size Cohen´s d based on differences between the intervention and control group; [b]Last 7 days
