# Supplementary material for: The Effectiveness of a Web-Based Self-Help Program to Reduce Alcohol Use Among Adults With Drinking Patterns Considered Harmful, Hazardous, or Suggestive of Dependence in Four Low- and Middle-Income Countries: Randomized Controlled Trial
Source: J Med Internet Res. 2021 Aug 27;23(8):e21686. doi: 10.2196/21686 (PMC8433861; doi:10.2196/21686)
Supplement: Multimedia Appendix 7 [file jmir_v23i8e21686_app7.pdf]

Multimedia Appendix 7. Detailed analysis for every study country.

	Brazil N = 587		Mexico N = 509		India N = 212		Belarus N = 92		Total N = 1400	
	**IG** n = 290	**CG** n = 297	**IG** n = 256	**CG** n = 253	**IG** n = 95	**CG** n = 117	**IG** n = 46	**CG** n = 46	**IG** n = 687	**CG** n = 713
Complete Case, N (%)	80 (27.6)	101 (34.0)	88 (34.4)	146 (56.9)	53 (55.8)	52 (44.4)	17 (37.0)	26 (56.5)	239 (34.8)	323 (45.3)
**AUDIT Baseline, M(SD)**	22.3 (6.8)	22.2 (6.5)	22.6 (6.3)	22.3 (6.8)	30.2 (7.2)	30.2 (8.6)	13.1 (4.1)	14.4 (5.7)	22.9 (7.5)	23.0 (7.9)
**AUDIT Follow-Up, M(SD)**	15.7 (8.9)	19.5 (8.7)	12.2 (7.9)	18.3 (8.3)	22.3 (7.2)	23.8 (9.7)	5.6 (2.6)	7.6 (6.2)	15.2 (9.1)	18.7 (9.3)
**AUDIT Change, M(SD)**	5.1 (8.4)	1.8 (6.6)	9.6 (8.4)	3.3 (7.2)	7.5 (5.7)	4.6 (8.2)	6.8 (3.3)	5.3 (6.2)	7.4 (7.8)	3.2 (7.1)
**Statistical Analysis**	B= -3.23, 95% CI: -5.42 to -1.03, *P* = .004, d=.43		B= -6.26, 95% CI: -8.30 to -4.22, *P* < .001, d=.82		B= -2.89, 95% CI: -5.61 to -0.18, *P* = .037, d=.41		B= -1.50, 95% CI: -4.79 to 1.80, *P* = .365, d=.30		B= -4.18, 95% CI: -5.42 to -2.93, *P* < .001, d=.56	
**Standard Drinks Baseline, M(SD)**	44.6 (29.6)	42.4 (28.6)	28.3 (18.4)	30.4 (19.6)	93.0 (71.2)	90.5 (69.1)	14.5 (14.4)	13.7 (12.4)	43.2 (41.1)	44.2 (41.7)
**Standard Drinks Follow-Up, M(SD)**	15.5 (19.5)	31.1 (29.6)	6.7 (8.9)	20.8 (25.7)	29.4 (17.7)	27.7 (16.0)	4.4 (4.1)	7.5 (11.8)	12.6 (16.3)	23.7 (26.3)
**Drinks Change, M(D)**	22.3 (28.3)	14.7 (32.5)	21.0 (20.0)	13.6 (19.6)	55.1 (85.3)	39.2 (52.4)	5.7 (4.4)	4.5 (6.1)	24.7 (39.6)	15.4 (28.4)
**Statistical Analysis**	B= -7.99, 95% CI: -18.56 to 2.58, *P* = .14, d=.26		B= -7.35, 95% CI: -12.98 to -1.72, *P* = .011, d=.37		B= -15.94, 95% CI: -60.81 to 28.93, *P* = .48, d=.44		B= -1.19, 95% CI: -0.67 to 0.51, *P* = .51, d=.23		B= -9.34, 95% CI: -15.92 to -2.75, *P* = .006, d=.28	
Imputed Data	**IG** n = 290	**CG** n = 297	**IG** n = 254	**CG** n = 253	**IG** n = 95	**CG** n = 117	**IG** n = 46	**CG** n = 46	**IG** n = 687	**CG** n = 713
**AUDIT Baseline, M(SD)**	22.3 (6.8)	22.2 (6.5)	22.6 (6.3)	22.3 (6.8)	30.2 (7.2)	30.2 (8.6)	13.1 (4.1)	14.4 (5.7)	22.9 (7.5)	23.0 (7.9)
**AUDIT Follow-Up, M(SD)**	17.1 (8.6)	19.9 (8.5)	13.5 (7.8)	18.8 (8.2)	NA	NA	NA	NA	15.5 (8.7)	19.8 (9.4)
**AUDIT Change, M(SD)**	5.1 (7.9)	2.3 (7.3)	9.1 (7.8)	3.5 (7.3)	NA	NA	NA	NA	7.3 (7.3)	3.3 (7.4)
**Statistical Analysis**	B= -2.82, 95% CI: -5.49 to -0.16, *P* = .039, d= .37		B= -5.54, 95% CI: -7.50 to -3.57, *P* < .001, d= .74		NA[1]		NA[2]		B = -4.08, 95% CI: -5.32 to -2.85, *P* < .001, d= .56	

**Standard Drinks Baseline, M(SD)**	44.8 (29.7)	42.9 (28.8)	28.7 (19.1)	31.7 (22.2)	90.7 (68.0)	85.9 (64.5)	12.3 (8.6)	11.8 (9.0)	43.3 (41.1)	44.6 (41.9)
**Standard Drink Follow-Up, M(SD)**	17.5 (20.8)	28.8 (26.5)	9.1 (12.2)	19.1 (21.9)	NA	NA	NA	NA	15.9 (20.3)	28.8 (32.0)
**Drinks Change, M(D)**	27.2 (29.9)	14.1 (32.1)	19.5 (17.9)	12.7 (19.4)	NA	NA	NA	NA	27.3 (37.5)	15.8 (39.0)
**Statistical Analysis**	B = -13.14, 95% CI: -23.34 to -2.94, *P* = .013, d= .42		B = -6.84, 95% CI: -11.01 to -2.68, *P* = .001, d= .37		NA[1]		NA[2]		B = -11.6, 95% CI: -17.19 to -6.02, *P* < .001, d= .30	

AUDIT = Alcohol Use Disorders Identification Test, [1] Questionable imputation model that cannot be improved, which is why the ITT specification was omitted here. The best model that we were able to calculate was also significant in the ITT analysis.
